# Overcoming the immune suppressive nature of glioblastoma by leveraging the surgical intervention - current status and future perspectives

**DOI:** 10.3389/fimmu.2023.1183641

**Published:** 2023-05-19

**Authors:** Johnny Duerinck, Sandra Tuyaerts, Kiavash Movahedi, Bart Neyns

**Affiliations:** ^1^ Department of Neurosurgery, Universitair Ziekenhuis Brussel (UZ Brussels), Brussels, Belgium; ^2^ C4N - Center for Neurosciences, Vrije Universiteit Brussel, Brussels, Belgium; ^3^ Laboratory for Medical & Molecular Oncology (LMMO), Vrije Universiteit Brussel, Brussels, Belgium; ^4^ Laboratory for Molecular and Cellular Therapy (LMCT), Vrije Universiteit Brussel, Brussels, Belgium; ^5^ Department of Medical Oncology, Universitair Ziekenhuis Brussel (UZ Brussels), Brussels, Belgium

**Keywords:** glioblastoma, immune therapy, immune checkpoint inhibition, local administration, surgery

## Abstract

Despite relentless efforts to improve outcome, the prognosis of glioblastoma (GBM) remains poor. Standard therapy at first diagnosis consists of maximal safe surgical resection followed by radiochemotherapy, but treatment options at recurrence are scarce and have limited efficacy. Immunotherapy is a broad term that covers several treatment strategies, including immune checkpoint inhibition (ICI). The successes of systemically administered therapeutic monoclonal antibodies that block the Programmed death receptor or ligand (PD-(L)1) and Cytotoxic T-Lymphocyte associated protein (CTLA)-4 immune checkpoints in other cancer types could not be reproduced in glioblastoma. This is considered to be related to the intrinsic low immunogenicity and strong immunosuppressive tumor microenvironment of glioblastoma, in addition to the presence of a blood-glioma and blood-brain barrier that limits many systemically administered therapeutic agents from reaching their target. In this mini-review, we address the specific aspects of immune suppression in glioblastoma and discuss potential strategies that could help to overcome it. The potential advantages of incorporating surgical resection in clinical trials of immunotherapy for glioblastoma, including window-of-opportunity studies, are highlighted. Combination strategies that include surgical resection, as well as local administration of therapeutic agents in the brain are discussed as a potential strategy to achieve an effective immunological response against glioblastoma.

## Introduction

Glioblastoma (GBM) is a deadly disease. Standard treatment consists of maximal safe resection followed by radiochemotherapy. This treatment prolongs survival but the tumor recurs in the vast majority of patients, at which point no effective standard therapy has been defined (1). If glioblastoma recurrence is amenable for surgery, surgical resection will be proposed to the patient, but it is generally accepted that this needs to be completed with systemic therapy. Tumor response rates of any salvage therapy (chemotherapy, targeted- or immunotherapy) are typically shown to be 5 to 10%, six-month progression-free survival (6mPFS) from recurrence onward around 15% (PFS of 2 to 4 months), and median overall survival (OS) around 25 weeks (2, 3). Despite disappointing success rates of clinical trials, most studies have shown that a small minority of the patients will have durable responses to certain study treatments before relapse occurs - not influencing median survival but forming the tail of the Kaplan-Meier survival curve. This implies that response to treatment can differ greatly between patients, and characterizing which specific features of the tumor predicts a better response to available treatment options could advance treatment success in selected glioblastoma patients significantly. Attempts at predicting response have been frustrating, however, and no predictive marker has been validated for clinical decision-making today. It is assumed that this would require more detailed knowledge of the different aspects of the tumor at the molecular level, and an understanding of how first-line treatment influences tumor evolution in space and time. Besides important intertumoral heterogeneity, glioblastoma also has significant intratumoral heterogeneity that pertains to the tumor cells as well as to the tumor microenvironment (TME). This heterogeneity is the result of differential selective pressures originating from cancer treatment as well as the anti-tumor immune response, which leads to resistant clones that drive tumor progression, contributing to the aggressive behavior and strong immunosuppressive nature of the disease (4). Immune therapy has proven successful in tumors with high mutational burden and an inflammatory TME such as melanoma and non-small cell lung carcinoma, but large trials of systemically administered immune checkpoint inhibitors (ICI) in glioblastoma have shown mainly negative results (5, 6). Even though there is no improved overall survival and response rates are below 10%, responses - when they occur - are characterized by dense immune-cell infiltration on histopathology and tend to be more durable than with other treatment types (7). Case reports of children with germline bi-allelic mismatch repair deficiency (MMRd)-related glioblastoma successfully treated with checkpoint inhibitors at least demonstrate that the immune system can be harnessed to be active against glioblastoma and that the main challenge lies in converting the strong immunosuppressive microenvironment from a “cold” into a ‘hot’ one (5, 7). In adult patients, however, high microsatellite unstable (MSI-H) glioblastoma,harbouring MMRd, was found to be refractory to treatment with pembrolizumab (8). Knowledge of the mechanism of immune evasion of glioblastoma is constantly increasing and provides insight into what could constitute an effective immunotherapeutic strategy to treat this tumor.

## The anti-tumor immune response

The current canonical concept of the cancer-immunity cycle dictates that tumor-specific antigens that are released from the tumor cells upon cell death are presented by dendritic cells (DCs) to the T-cells, leading to their activation and clonal expansion (9). Immune checkpoint signaling is crucial to regulate immunological inhibitory feedback mechanisms and the activation of T-cells (10). The activated T-cells then proceed to attack tumor cells expressing these antigens, causing more immunogenic cell death (ICD) (9). If, however, the immune response is not successful in completely eliminating the threat, natural selection of immune-resistant tumor cells is promoted. This process is known as immuno-editing and may select for immunosuppressive escape mechanisms (11). Based on the quantification of CD3 and CD8-positive T lymphocytes both in the tumor center and the invasive margin (ie the Immunoscore), tumors can be classified as hot, immunosuppressed, T-cell excluded, or cold (12, 13). These subtypes typically also harbor differential expression of immune checkpoint molecules, immune signature in gene expression analysis, and tumor mutational burden. Tumors are considered hot when they harbor high T-cell infiltration, expression of Programmed death-ligand 1 (PD-L1), high tumor mutational burden (TMB), and increased Interferon-gamma signaling. Immunosuppressed tumors show an intermediate amount of infiltrating T cells but have a TME that limits activation and further recruitment of immune cells despite the absence of physical barriers. Tumors that are characterized by poor T cell infiltration, low TMB, low Major Histocompatibility Complex (MHC) expression, and low PD-L1 expression are considered cold. As a fourth subtype, in excluded tumors, T cells and other effector cells are unable to efficiently infiltrate into the tumor nests and instead accumulate at the invasive margins of the tumor. Excluded tumors typically display increased hypoxia and increased angiogenesis, hallmarks of glioblastoma.

## Immune suppression in glioblastoma

The glioblastoma TME is characterized by a high concentration of several immunosuppressive molecules, such as Interleukin (IL)-6, IL-10, Transforming Growth Factor-beta (TGF-β), and prostaglandin E2 (PG-E2). These molecules are produced by the tumor cells and tumor-associated macrophages (TAMs). TAMs form the largest immune infiltrate in glioblastoma tumors and exhibit extensive heterogeneity, reflecting their intrinsic ability to respond to the various environmental cues that exist within the tumor microenvironment. TAMs were found to consist of microglia (Mg)- and monocyte (Mo)-derived populations, with both TAM subsets displaying varying, but mostly tumor-promoting activities (14–17). Inhibition of the anti-tumor immune response by TAMs and tumor cells occurs by downregulating MHC expression, suppressing T-cell activation and expansion, inducing T-cell anergy, activating suppressive regulatory T-cells (Treg) and myeloid-derived suppressor cells (MDSC), and driving TAMs to the immunosuppressive M2 phenotype (18). Due to the aggressive growth of glioblastoma, tissue hypoxia is a frequent phenomenon. The hypoxia induces activation of HIF-1alpha and eventually VEGF production which has immunosuppressive properties (19).

As a result of the low tumor mutational burden (TMB) combined with all these mechanisms of immune suppression resulting in a relative paucity of tumor-infiltrating lymphocytes and low expression of anti-PD1, GBM has typically been labeled as a cold tumor. Even though all GBMs generally harbor an immune suppressive TME, recent work has shown inter-tumor heterogeneity in the immune contexture of glioblastoma TME. By studying the IDH-wt GBM TME transcriptome, three distinct GBM subtypes have been revealed: TMEhigh, TMEmed, and TMElow (20). The TMEhigh tumors have significantly increased expression of genes specific to all immune populations (elevated lymphocyte, myeloid cell immune checkpoint, PD-1, and CTLA-4 transcripts), TMEmed tumors are enriched for endothelial cell gene expression profiles and show heterogeneous immune cell populations. TMElow tumors show low expression of all immune and endothelial cell markers and are thus considered an ‘immune desert’ group.

Even though the mesenchymal subtype relatively less often has TMElow immune composition (10% vs 35% and 55% for the proneural and classical subtype, respectively), all TME subtypes could be found in all tumor cell gene expression subtypes (21). No correlation was observed between the specific TME subtype and patient survival. Nonetheless, response to immune therapy seemed better in the TMEhigh group of patients (20). This study also showed that these novel TME subtypes are dynamic and tend to evolve across primary and recurrent GBMs, contributing to longitudinal heterogeneity. This could imply that there is the potential for a forced subtype switch in glioblastoma, making the tumor more responsive to immune therapy. Either way, each TME subtype most likely requires a different immune strategy in order to optimize response (22).

## Overcoming the immune suppressive nature of glioblastoma

Systemic administration of ICI has not been successful in obtaining improved survival results for patients with glioblastoma. ( (5, 6) It has been suggested before that for tumors with a cold TME, which by definition have deployed a combination of immunosuppressive strategies, combinatorial therapeutic strategies need to be deployed to achieve tumor response. This includes combining immune therapies (vaccination therapy, adoptive cell transfer, oncolytic virus therapy, ICI, TAM reprogramming) but also looking at smart immunotherapy combinations with currently used treatments such as surgery and radiation therapy (23). Indeed, these commonly employed treatments not only lead to tumor mass reduction and depletion of immunosuppressive cells, but also to increased tumor antigen uptake and presentation by DCs, thus promoting anti-tumor immunity (22). The exact effect of temozolomide (TMZ)-chemotherapy on immunotherapy effectiveness has not been fully elucidated. Although it has been suggested that lymphopenia caused by TMZ could prove beneficial since eradication of immune suppressive cells takes place, recent studies rather show that TMZ exerts a negative effect on immune effector cells (24). The immunosuppressive effect of corticosteroids, which are frequently used to reduce peritumoral edema in GBM patients, has been well established, and it is generally advisable to attempt to withhold steroids from patients treated with immunotherapy (25).

Vascular endothelial growth factor (VEGF) has been demonstrated to suppress antitumor immunity by stimulating the proliferation of regulatory T-cells (Treg) and inhibiting Dendritic Cell (DC) maturation (26, 27). Antiangiogenic agents that target VEGF or its receptor have been proposed as the ideal adjunct to immune therapy. This is not only because they could reinforce the antitumor immune response but also because they are effective against peritumoral edema and thus can limit the necessity of immunosuppressive corticosteroids. However, studies that combined the small molecule VEGF-R Tyrosin Kinase Inhibitor (TKI) axitinib with the anti-PD-L1 ICI avelumab or the monoclonal anti-VEGF antibody Bevacizumab with the anti-PD1 ICI pembrolizumab have not been able to demonstrate improved overall survival (28, 29). This might be due to the ineffectiveness of systemically administered ICI, and should not rule out antiangiogenic agents as a potential adjunct to effective immunotherapy for glioblastoma.

The use of ICI prior to surgery (neoadjuvant treatment) has been proposed as a promising strategy. In patients with recurrent glioblastoma, neoadjuvant anti-PD1 therapy (with nivolumab or pembrolizumab) resulted in higher immune cell infiltration, enhanced expression of interferon-γ-related genes, and enhanced clonal expansion of T cells (30, 31). Even though the impact of this neo-adjuvant therapy on survival is still not established, the principle that neo-adjuvant therapy with PD1 blockade induces a local immunomodulatory effect has been demonstrated.

## Combination of surgery and immunotherapy

The role of the surgical intervention in the treatment of glioma of any type is currently being revalued in itself since it has been demonstrated that supratotal resection for low-grade glioma but also for glioblastoma improves survival compared to total resection (32, 33). This suggests that the resection of the invasive front of the tumor, which in glioblastoma is most likely located in the non-contrast enhancing area of the tumor, yields additional benefit.

The net immunological effect of the surgical resection is leaning toward immune suppression. Although the inevitable tissue and vascular trauma caused during surgery triggers the innate and, at a later stage, the adaptive immune system, regulation by immune suppression quickly follows. This immune suppression is characterized by the release of growth factors (VEGF, Platelet-derived growth factor (PDGF), TGF-β), clotting factors, stress hormones (glucocorticoids, catecholamines, prostaglandins), and cytokines into the extracellular space, and tends to last several weeks following the initial cancer surgery (34, 35). The postoperative period is thus marked by an immune suppressive state but there is mounting evidence that this state is reversible (34). Moreover, specifically in glioblastoma, the debulking of the hypoxic core and invasive margin of the tumor may achieve a reduction of immunosuppression as a net result. Consequently, the surgery and perioperative period provide a window of opportunity that not only can be exploited to obtain tissue for analysis but also to reduce immunosuppression and mount an effective anti-tumor immune response.

## Local administration of immunotherapeutic agents

The search for effective systemic therapy for glioblastoma has not only been troubled by the intrinsic therapeutic resistance mechanisms and substantial spatial and longitudinal heterogeneity of these tumors, but also by the fact that they lie secluded within the confines of the Blood-Brain Barrier (BBB) (36). Since the BBB prevents most systemic agents from reaching their target within the central nervous system, local delivery strategies are increasingly being explored. Explored strategies for local application include drug-loaded biodegradable wafers, drug-loaded hydrogels, intrathecal delivery using an Ommaya reservoir, direct intraoperative injection into the brain tumor or resection cavity wall and convection-enhanced delivery through one or several implanted catheters (37–39). Each of these strategies has its specific advantages and disadvantages (40). Local administration of recombinant oncolytic virus (recombinant polio–rhinovirus chimera PVSRIPO, adenovirus DNX-2401, Vocimagene Amiretrorepvec TOCA-511, etc) has been performed previously (41–43) Direct local injection of the therapeutic agent has the advantage that it is straightforward, can be incorporated into the workflow of a brain tumor resection, and can be performed at multiple locations allowing coverage of the entire tissue lining of the resection cavity.

## Glitipni trial

**Figure 1:**
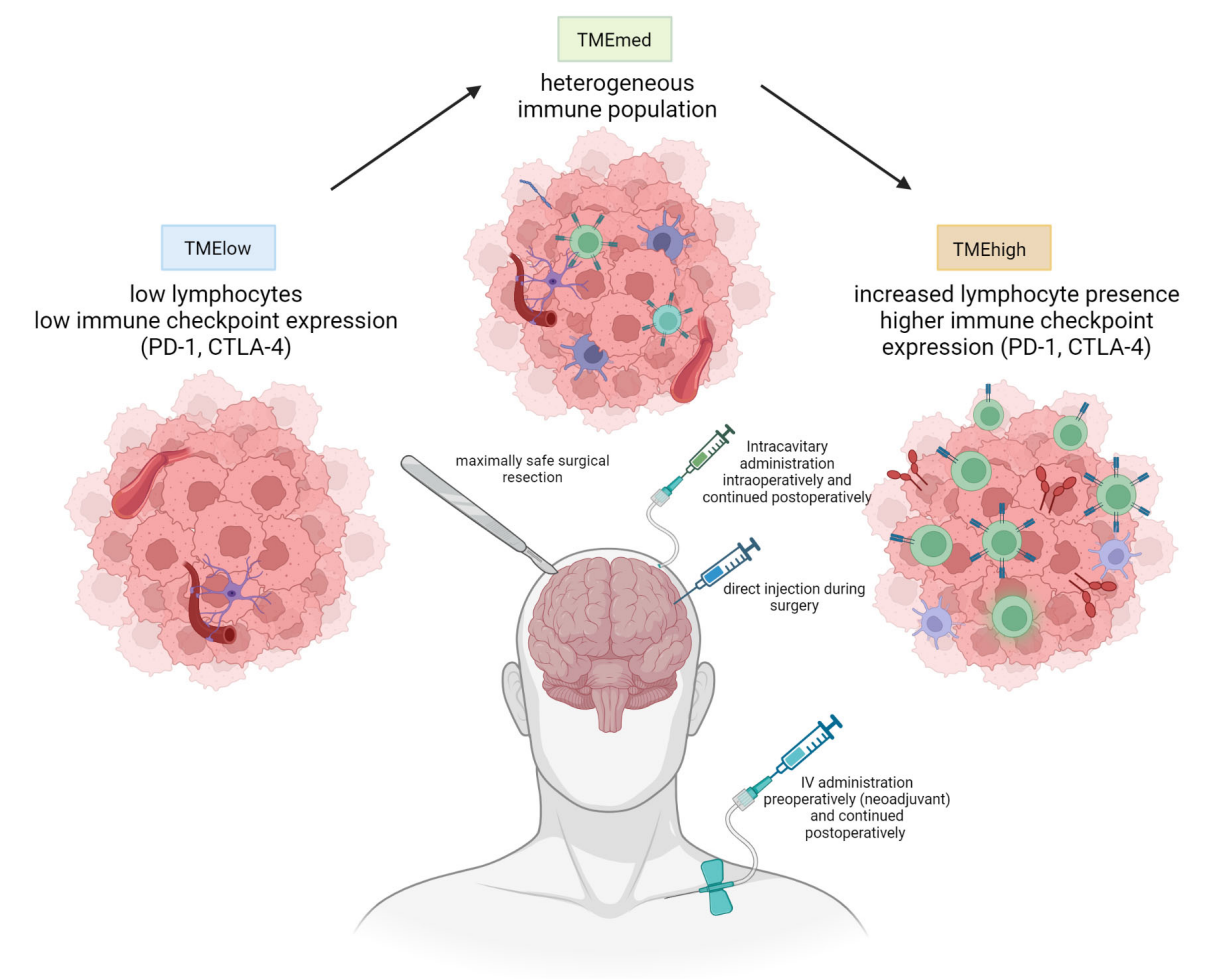
Current methods used in the Glitipni study. The aim of the strategy is to combine surgical treatment with a combination of local and systemic immunotherapy by decreasing immunosuppression and inducing immune response. Currently employed immunotherapeutic agents in our trials include local, locoregional, and systemic immune checkpoint inhibitors and local myeloid dendritic cells (created with BioRender.com).

In order to obtain a sufficiently high local concentration of nivolumab and ipilimumab while still avoiding potential (systemic) side effects that have traditionally limited clinical trials with anti-cytotoxic T-lymphocyte associated protein-4 (CTLA-4), we hypothesized that local administration following resection of glioblastoma could improve the anti-tumor immune response and improve patient outcome (44). We thus set up a phase I adaptive clinical trial (the “Glitipni” trial) with intracerebral injection of nivolumab and ipilimumab where, after dose escalation for each product has been performed, additional methods of local administration as well as additional immunotherapy are to be added to new cohorts. Patients with recurrent glioblastoma that are candidates for resection of their recurrence, have good performance status, and are not taking steroids are eligible for participation. Twenty-four hours preoperatively, patients receive an intravenous administration of low-dose (10mg) nivolumab. Maximal safe resection is performed, and at the end of the surgery, the immune checkpoint inhibitors (ipilimumab (IPI) 10mg/2ml alone in cohort 1 and ipilimumab 5mg/1ml and nivolumab 10mg/1ml from cohort 2 onward) are injected in the wall of the resection cavity in 20 to 30 injections of approximately 0,1ml each. (**Figure 1**) Postoperatively, intravenous administration of low-dose nivolumab was pursued every two weeks. These methods were used in cohorts 1 and 2, the results of which have been published recently and are summarized hereunder (45).

## Patient demographics

Twenty-seven patients in total were enrolled: three patients were included in cohort 1, and twenty-four in cohort 2. Most patients (81%) had a World Health Organisation (WHO) grade IV tumor at first diagnosis and 93% had an Isocitrate dehydrogenase (IDH)-wild-type tumor. All patients had surgical resection at first diagnosis, followed by standard radiochemotherapy in 85%. Thirty percent of patients had previously undergone resection for recurrence at inclusion, and the median number of prior systemic therapies was three. Summarizing, these two cohorts generally consisted of patients with good performance scores, under low-dose or no corticosteroids but with some history of treatment for recurrence.

## Adverse events

There were no surgical complications related to the intraoperative injections of ipilimumab (cohort-1), or ipilimumab plus nivolumab (cohort-2). Postoperative fever was seen in 14/27 patients and was grade 1 in 12 patients, which included all three patients from cohort-1, and grade 2 in 2 patients. There was a low incidence of suspected immune-related adverse events: pruritus (n=7), rash (n=4), hypothyroidism (n=2), a sarcoid-like reaction (n=1), and fatigue (n=17). Subacute postoperative neurological deterioration was seen in two patients. This was considered to be due to excessive edema as a result of the immune response, and full recovery was seen in these patients upon steroid treatment.

## Efficacy

The median PFS for both cohorts combined was 11.7 weeks (95% CI: 10 to 12; range 2–152), while median OS was 38 weeks (95%CI: 27 to 49) with a one and two-year PFS of 40.7% (95% CI: 22 to 59) and 27% (95% CI: 9 to 44), respectively. Proof-of-concept of an effective immune response following study treatment was seen in the three patients that underwent repeat resection upon suspected recurrence. In one of these patients, the resected tissue consisted of glioblastoma tumor cells with focal lymphocytic infiltration. In the two other patients, the resected tissue consisted of infiltration by inflammatory cells mainly composed of lymphocytes without any evidence of glioblastoma tumor recurrence at all. In translational analyses, no correlation could be found between PD-L1 immunohistochemistry or gene expression profile and OS in univariate analysis. B7-H3 gene expression was the only factor that remained associated with significantly worse survival in multivariate Cox logistic regression (p>0.029).

## Current status and future prospects for Glitipni

As illustrated in **Table 1**, adaptations to dosing, administered compounds, and methods of administration have been made following the first two cohorts. At the moment of writing, 102 patients have been treated in the context of the trial, spanning all cohorts. Results from grouped cohorts will be published in the following year.

**Table 1:** Overview of cohorts in the Glitipni trial.

Cohort	Additional eligibility criteria	Recruitment Status	No. of patients	Study Treatment			Recruitment period						
				Per-operative		Post-operative*	2017	2018	2019	2020	2021	2022	2023
1	Resectable	Completed	3	MSR	iCer 10 mg IPI	IV 10 mg NIVO Q2w x6	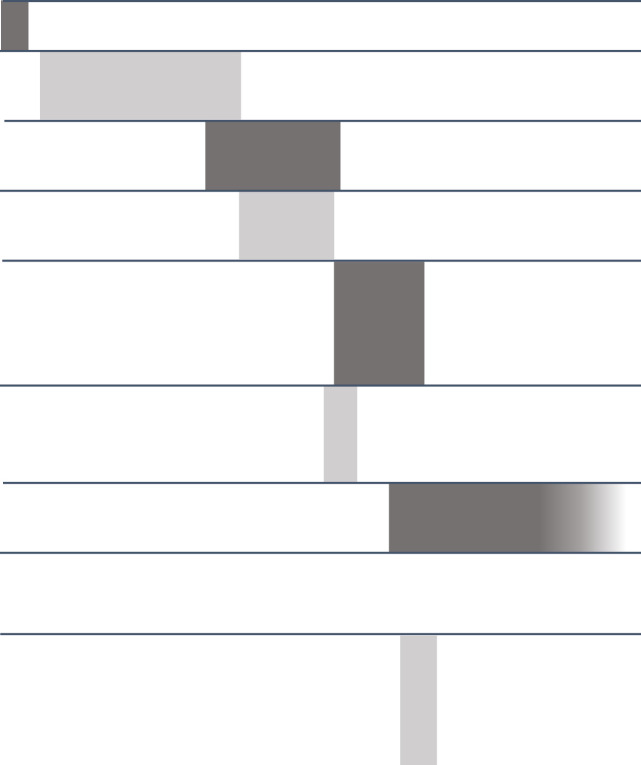						
2		Completed	24	MSR	iCer 5 mg IPI + 10 mg NIVO								
3	Unresectable	Completed	16	STx	iTum 5 mg IPI + 10 mg NIVO	IV 10 mg NIVO + iCav 1/5/10 mg NIVO Q2w x12							
4	Resectable	Completed	16	MSR	iCer 5 mg IPI + 10 mg NIVO								
5	Resectable	Paused	11	MSR	iCer 5 mg IPI + 10 mg NIVO iCer myDC (1- 10- 20.10^6^)	IV 10 mg NIVO + iCav 10 mg NIVO Q2w x12							
6	Unresectable	Discontinued	2	STx	iTum 5 mg IPI + 10 mg NIVO iTum myDC								
7	Resectable	Ongoing	27+	MSR	iCer 5 mg IPI + 10 mg NIVO	IV 10 mg NIVO + iCav 10 mg NIVO + iCav 1/5/10 mg IPI Q2w x12							
8	Unresectable	Canceled	0	STx	iTum 5 mg IPI + 10 mg NIVO								
9	Unresectable	Discontinued	3			IV 10 mg NIVO + iThe 10 mg NIVO + iThe 1/5/10 mg IPI Q2w x12							

In the cohorts following cohort 1 and 2, an Ommaya reservoir was also implanted at the end of the surgery with the catheter hanging freely in the resection cavity, allowing to additionally perform intracavitary injections of nivolumab (cohorts 3 to 6) and the combination of nivolumab and ipilimumab (cohort 7). Cohorts 3, 6, and 8 allowed for the inclusion of patients with unresectable recurrent glioblastoma but with a preexisting resection cavity. Local injection in these patients was performed by stereotactic needle. In cohorts 5 and 6, BDCA1+ and BDCA3+ positive myeloid dendritic cells, obtained through leukapheresis, were injected intracerebrally at the end of the surgery, in addition to ipilimumab and nivolumab. From cohort 3 onwards, cerebrospinal fluid from the peritumoral area is obtained every two weeks, allowing cytological and also pharmacological analysis. MSR, Maximally Safe Resection; STx, stereotactic biopsy; iCer, intracerebral injection; iTum, intraTumoral injection; iCav, intracavitary injection; NIVO, Nivolumab; IPI, Ipilimumab.

Thus far, we performed experimental therapy with intracerebral and intracavitary injection of ICI in varying combinations and administration routes, and also combined ICI with intracerebral injection of autologous myeloid dendritic cells. In earlier work performed by our group, intratumoral injection of these cells was found to be able to obtain durable complete remissions in some patients with ICI refractory melanoma (46). For the planned upcoming trial, we will complement the established per- and postoperative immunotherapy with neoadjuvant administration of ipilimumab and nivolumab starting four weeks prior to the surgical intervention, at which point intracerebral and intracavitary injection of ICI will take place at the doses established in the previous cohorts. This will also allow us to sample Cerebrospinal fluid (CSF) and tissue to examine the concentration of study drugs. Extensive translational analysis of the tumor tissue and CSF, including bulk and single-cell tumor and TME genome profiling, as well as assessment of the T-cell repertoire, will also allow us to look into the impact of neoadjuvant therapy on the TME and help in developing predictive markers for the effectiveness of immunotherapy.

## Conclusions

Many hurdles remain in finding an effective treatment for glioblastoma, but preclinical and clinical evidence points towards a potentially important role for immunotherapy. Difficulties remain determining the optimal method of administration, choosing the exact immunotherapy type, personalization of therapy to suit the patient’s tumor-specific characteristics, and judging the role of the combination of immunotherapy with currently employed treatment, including surgery, chemo- and radiotherapy, and perhaps even steroids. In immunologically cold tumors like glioblastoma, combinations of immunotherapeutic efforts are most likely to generate an effective anti-tumor immune response. Administering ICI in the neo-adjuvant setting prior to surgery seems to shift the tumor TME towards a more immunotherapy-receptive environment in a subset of cases. The surgical intervention following the neo-adjuvant treatment can also be leveraged to not only locally administer the immunotherapeutic agents but also to obtain tissue for pharmacokinetic studies. As a locally administered immunotherapeutic, the combination of ICI with myeloid dendritic cells currently seems most promising based on the preliminary results of our Glitipni clinical trial.

## Author contributions

JD wrote the first draft of the manuscript. All authors contributed to manuscript revision, read, and approved the submitted version.
